# Development and characterization of monoclonal antibodies against the extracellular domain of African swine fever virus structural protein, CD2v

**DOI:** 10.3389/fmicb.2022.1056117

**Published:** 2022-11-18

**Authors:** Siyuan Liu, Peiyang Ding, Yongkun Du, Dongna Ren, Yilan Chen, Minghui Li, Xueke Sun, Siqiao Wang, Zejie Chang, Ruiqi Li, Gaiping Zhang

**Affiliations:** ^1^College of Animal Medicine, Henan Agricultural University, Zhengzhou, China; ^2^Henan Provincial Key Laboratory of Animal Immunology, Henan Academy of Agricultural Sciences, Zhengzhou, China; ^3^College of Life Sciences, Zhengzhou University, Zhengzhou, China; ^4^Jiangsu Co-innovation Center for Prevention and Control of Important Animal Infectious Diseases and Zoonoses, Yangzhou University, Zhengzhou, China

**Keywords:** African swine fever virus, CD2v protein, monoclonal antibodies, B-cell epitope, extracellular

## Abstract

African swine fever virus (ASFV), a DNA double-stranded virus with high infectivity and mortality, causing a devastating blow to the pig industry and the world economy. The CD2v protein is an essential immunoprotective protein of ASFV. In this study, we expressed the extracellular region of the CD2v protein in the 293F expression system to achieve proper glycosylation. Monoclonal antibodies (mAbs) were prepared by immunizing mice with the recombinant CD2v protein. Eventually, four mAbs that target the extracellular region of the ASFV CD2v protein were obtained. All four mAbs responded well to the ASFV HLJ/18 strain and recognized the same linear epitope, ^154^SILE^157^. The specific shortest amino acid sequence of this epitope has been accurately identified for the first time. Meaningfully, the ^154^SILE^157^ epitope was highly conformed in the ASFV Chinese epidemic strain and Georgia2008/1 strains according to the analysis of the conservation and have a fair protective effect. These findings contribute to further understanding of the protein function of CD2v and provide potential support for the development of diagnostic tools and vaccines for ASFV.

## Introduction

The African swine fever virus (ASFV) is the origin of the infectious illness known as African swine fever (ASF), which causes acute hemorrhagic sickness (Franzoni et al., 2018). ASF was listed as a Class I animal illness by the People’s Republic of China’s Ministry of Agriculture in December 2018 (Announcement No. 1125 of the Ministry of Agriculture of the People’s Republic of China). It is categorized as a legally reported animal illness by the World Organization for Animal Health [Office International des épizooties (OIE); Zhou et al., 2018].^1^ ASFV was initially discovered and isolated in Kenya, Africa, in 1921, and it rapidly spread over the continent, killing the domestic pigs who came into contact with it (Sánchez-Vizcaíno et al., 2012). ASF did not extend of the African continent to Portugal and Europe until 1957 (Sánchez-Cordón et al., 2018, 2019). The first ASF infection case in China was documented in 2018 and it occurred in Shenyang (Wang et al., 2018). Comparing the isolated strain’s conserved P72 gene to that of the Georgia strain revealed a perfect match (Bao et al., 2019). Since that time, ASF has swiftly expanded throughout China. The domestic swine breeding business in China has suffered a practically devastating blow because of the extraordinary number of domestic pigs that have passed away to date. China is the world’s largest producer and consumer of pork. Currently, the ASF epidemic remains severe (Zhou et al., 2018).

Since the ASFV epidemic, researchers have been looking into the virus’s pathogenic mechanisms and practical preventative measures (Sánchez-Vizcaíno et al., 2012; Brown and Bevins, 2018; Sánchez-Cordón et al., 2018). Currently, there is no effective vaccine to prevent ASF in domestic swine (Cubillos et al., 2013; Teklue et al., 2020). The genomic size of the long-strand DNA virus ASFV ranges from 170 to 190 KB. It is the sole member of the Asfarviridae family and a member of the NCLDV family, or nucleoplasmic large DNA viruses. ASFV encodes 150–200 proteins with 151–167 open reading frames (ORFs), 68 of which are structural proteins, and the remaining are nonstructural proteins (Gaudreault and Richt, 2019; Bosch-Camós et al., 2020). The multiple-layer structure of the ASFV virion has been identified as a regular icosahedron with the following layers: the nucleoid, core-shell, inner envelope, capsid, and outer envelope (Cubillos et al., 2013; Bosch-Camós et al., 2020; Teklue et al., 2020). The majority of the genes and proteins in the ASFV virion still have unknown roles, while the virion’s structure has been figured out. In order to develop effective ASF vaccines and establish diagnostic testing, it is crucial to examine the structural proteins and genes of the ASFV (Gaudreault and Richt, 2019; Sang et al., 2020).

The CD2v protein is encoded by the EP402R gene and named CD2v because of its high similarity to the T-cell surface binding receptor cluster differentiation 2 (CD2; Wang et al., 2019). CD2v is a transmembrane protein consisting of a signal peptide region, an extracellular domain (containing two immunoglobulin-like regions), a transmembrane region, an acidic domain, and a proline-rich domain (Alonso et al., 2018). According to several research, the CD2v protein is a crucial protective antigenic protein in ASFV. As a result, it may be a potential target in the development of vaccines (Liu et al., 2019). For instance, following immunization with CD2v-expressing pseudovirus, the mice were totally protected (Feng et al., 2020). Moreover, the CD2 V-deficient strain’s pathogenicity was dramatically decreased, showing that CD2v showed efficient immunogenicity (Goatley and Dixon, 2011; Arias et al., 2017; Jia et al., 2017). CD2v is also an important protein that is involved in ASFV infection and invasion (Frączyk et al., 2016). There are significant antigenic binding sites in the extracellular domain of CD2v, as shown by the identification of two immunoglobulin-like domains in earlier research as crucial regions for red blood cell (RBC) adherence and immunogenicity (Monteagudo et al., 2017). It was shown that the CD2v amino acid sequence is not the same across various ASFV strains, which may account for the debate about the protectiveness of the CD2v antigen (Feng et al., 2020). As a result, the identification of CD2v extracellular epitopes may be valuable in assisting in the diagnosis of various strains.

In this study, the CD2v extracellular domain that was expressed using the 293F mammalian expression system was selected as the immunogen to prepare mAbs (Borca et al., 1998). Eventually, four mAbs were obtained, and these mAbs reacted well with 293 T cells that were transfected with the EP402R gene. Results from IPMA (immunoperoxidase monolayer assay) showed that the four mAbs could specifically bind to the ASFV HLJ/18 strain. The mAbs identified the same linear B-cell epitope, 154SILE157 in Dot-Blot, Elisa and IFA tests. These results contribute to further elucidation of the function of CD2v and further study of ASFV, providing support for the development of diagnostic methods and vaccines.

## Materials and methods

### Cells and virus

Mouse Hybridoma (SP2/0) cells were obtained from ATCC (Manassas, VA, USA) and maintained in Roswell Park Memorial Institute medium (RPMI Medium 1640; Solarbio, Beijing, China) supplemented with 10% (v/v) fetal bovine serum (FBS, Trans, Beijing, China). Human embryonic kidney 293 T (HEK293T) cells were obtained from ATCC (Manassas, VA, USA) and maintained in Dulbecco’s modified Eagle’s medium (DMEM; Solarbio, Beijing, China) supplemented with 10% (v/v) fetal bovine serum (FBS, TransGen, Beijing, China). Human embryonic kidney 293F (HEK293F) cells were preserved by Henan Provincial Key Laboratory of Animal Immunology, Henan Academy of Agricultural Sciences (Zhengzhou, China). *Escherichia coli* Trans1-T1 Phage Resistant competent cells were purchased from TransGen Biotech (Beijing, China). The plates infected with the ASFV HLJ/18 strain were kindly provided by Professor Chen Weiye from Harbin Veterinary Research Institute, Chinese Academy of Agricultural Sciences. ASFV-positive sera (convalescent sera from surviving pigs that were naturally infected with ASFV) were a kind gift from Institute of Military Veterinary Medicine, Academy of Military Medical Sciences, Changchun, China.

### Recombinant ASFV CD2v extracellular domain protein was expressed in the 293F eukaryotic system

The extracellular domain sequence (CDS) of CD2v/EP402R gene based on ASFV-SY18 isolates (GenBank accession MH766894) was retrieved from the National Center for Biotechnology Information (NCBI) Reference Sequence database. Next, specific primers were designed to introduce EcoRI and XhoI [New England Biolabs (NEB), United States] restriction sites Ligated with pCAGGS vector (TransGen Biotech, China), correct recombinant gene was confirmed by DNA sequencing [Sangon Biotech (Shanghai) Co., Ltd., China], and thus CD2v recombinant protein was correctly constructed. The transient transfection of CD2v recombinant DNA was performed with Lip2000 (Gibco, ThermoFisher Scientific, United States) in HEK293F cells. At 72-h post transfection, successful expressions were examined by western blot analysis using ASFV positive sera. To purify recombinant CD2v proteins, HEK293F cell supernatants were collected and then purified by HiPrep Q FF column (GE Healthcare, United States). Thereafter, the purified recombinant CD2v proteins were resuspended in 20 mM Tris–HCl and 150 mM NaCl The protein was verified by western blotting with ASFV-positive serum.

### Monoclonal antibody generation and purification

**Figure 1:**
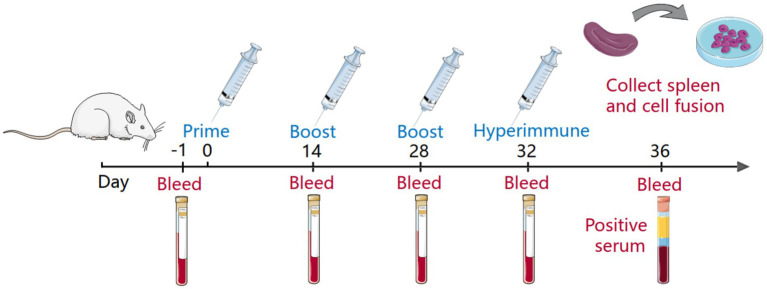
Immunity of mice and fusion of cells. The mice blood was collected as a negative serum before the primary immunization of the mice, and the serum titer was determined before each subsequent immunization. The immunity cycle of the mice was 21 days, and the mice underwent four immunizations, one primary immunization, two booster immunizations and a hyperimmunization before cell fusion. In addition, the serum before hyperimmunization was collected as positive serum.

Monoclonal antibodies against the extracellular domain of the CD2v protein were developed according to a previously reported method (Figure 1). In brief, BALB/c mice aged 6–8 weeks were subcutaneously immunized with 20 μg CD2v recombinant protein. The protein is emulsified by Freund’s complete adjuvant prior to immunization. Booster immunizations were performed 21 and 42 days after the primary immunization with the same dose of antigen emulsified with Freund’s incomplete adjuvant. Serum samples were collected at 21, 42 and 63 days, and the serum titer was determined by ELISA. The mouse with the highest antibody potency is injected intraperitoneally with 20 μg of CD2v recombinant protein. After an interval of 3 days, spleen cells from the mice were fused with mouse myeloma (SP2/0) cells. Hybridoma cells were selected in hypoxanthine-aminopterin-thymidine (HAT) medium. Finally, hybridoma cells were screened by indirect ELISA and subcloned more than 3 times by the limited dilution method. Positive hybridoma cell lines were injected into BALB/c mice to collect ascites fluid. Monoclonal antibodies were further purified from ascites fluid using HiTrap Protein G HP (GE Healthcare, United States).

### Monoclonal antibody characterization and identification

The reactivity between the monoclonal antibodies and CD2v recombinant protein was verified by Western blotting (WB). The ability of these monoclonal antibodies to bind the ASFV HLJ/18 strain was evaluated by an immunoperoxidase monolayer assay (IPMA). Briefly, 96-well plates seeded with primary porcine alveolar macrophages (PAMs) were infected with the ASFV HLJ/18 strain. The primary antibody was the supernatant of hybridoma cells. The secondary antibody was HRP-labeled goat anti-mouse IgG. After staining with a 3-amino-9-ethylcarbazole (AEC) solution (Solarbio, Beijing, China), the cells in 96-well plates were observed under light microscopy. The identification of mAbs was verified by indirect immunofluorescence assay (IFA). CD2v extracellular domain full-length plasmids were transfected into 293 T cells. The primary antibody was the supernatant of hybridoma cells. The secondary antibody was Alexa Flour 647-labeled goat anti-mouse IgG. The cells in 96-well plates were stained with a 4′,6-diamidino-2-phenylindole (DAPI) solution for 10 min and were observed under a fluorescence microscope. The mAb subtypes were determined by a mouse mAb subtype identification kit (Proteintech, Wuhan, China). The blocking-Elisa assay is following: each well was incubated with 50 μl diluted ASFV positive serum for 30 min at 37°C, then diluted CD2v mAbs were added and incubated for 30 min at 37°C. Followed, HRP conjugated goatanti-mouse IgG was incubated, and results were read at optical density of 450 nm. The percent of inhibition, [(OD450 value of negative controls − OD450 value of sample)/OD450 value of negative controls] × 100%.

### Identification of the antigenic epitopes

**Figure 2:**
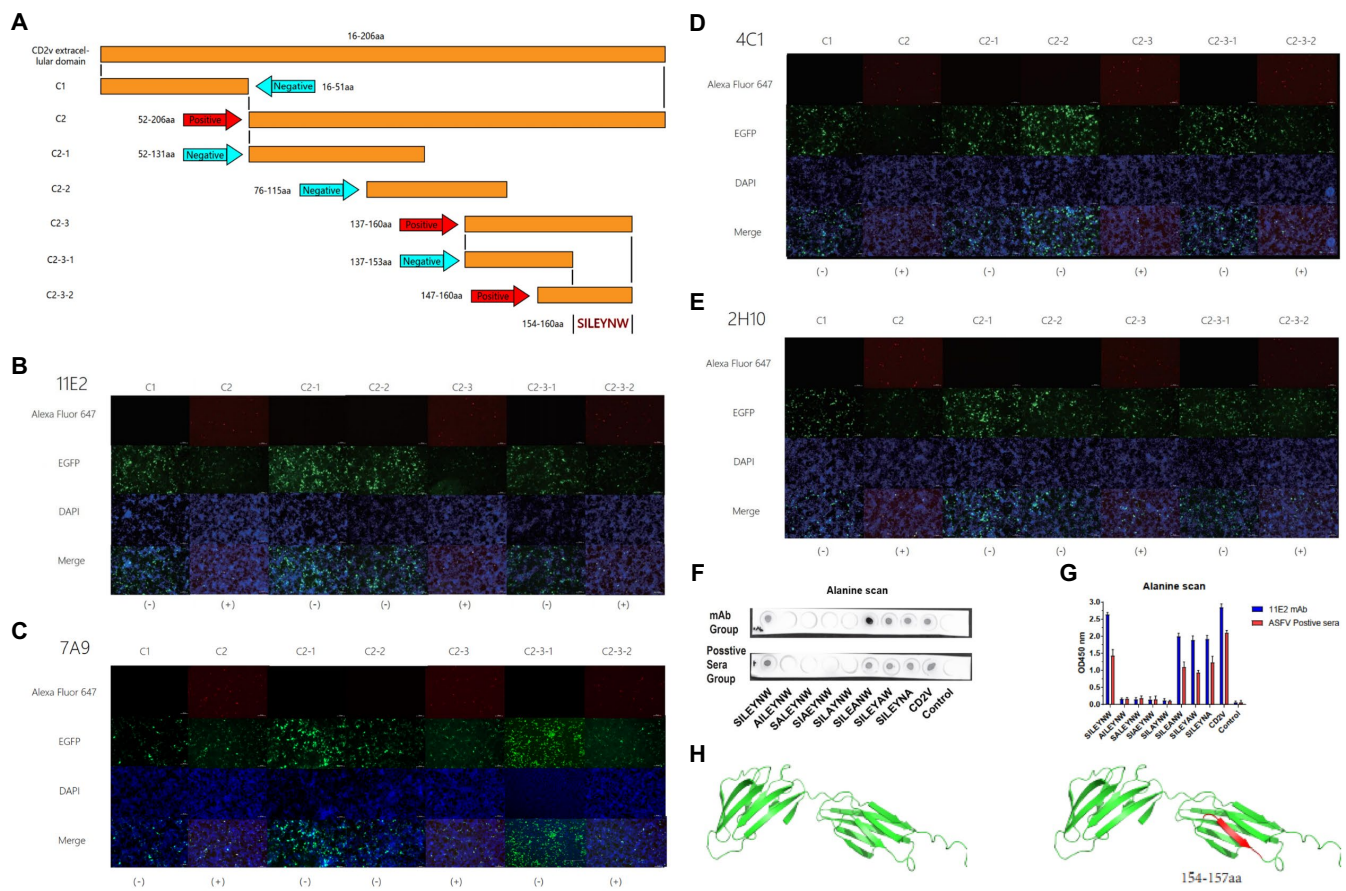
Mapping of critical binding epitope by mAbs and simulation of CD2v protein. **(A)** Design and presentation of amino acid sequences. **(B–E)** The binding of epitopes to mAbs was identified by IFA. **(B–E)** of 11E2, 7A9, 4C1 and 2H10 reacted with truncated sequences for epitope identification. (+) is a positive reaction. (−) indicates a negative reaction. **(F)** Dot-blot **(G)** Peptide-based ELISA. Identification of core residues. **(H)** Simulation of the epitope position in the CD2v protein. Epitope was marked by red.

The CD2v extracellular domain was truncated into C1 and C2 segments. In order to identify the binding sites of MAbs, the gene sequences of C1 and C2 were further truncated into five segments: C2-1, C2-2, C2-3, C3-1, and C3-2 (Figure 2A). The truncated gene sequence was amplified by PCR with designed primers and then ligated to the EGFP-C vector (TransGen Biotech, China). The recombinant plasmid was transfected into 293 T cells. The reactivity between the mAbs and the truncations were determined through IFA assay. The primary antibody was the supernatant of hybridoma cells. The secondary antibody was Alexa Flour 647-labeled goat anti-mouse IgG. The cells in 96-well plates were stained with 4′,6-diamidino-2-phenylindole (DAPI) solution for 10 min and observed under a fluorescence microscope.

To further identify the key amino acids at the binding site of the MAbs, the Dot-Blot and peptide-based Elisa experiments were performed according to the previously described methods (Jiang et al., 2020). The process is the peptide synthesis and alanine scanning of the amino acid sequence 154SILEYNW160 identified by IFA assays in the previous step. Here, the carrier protein (Bovine Serum Albumin, BSA) was coupled to the capture peptides for detecting and defining the epitopes to assure improved and directed attachment onto the support with SMCC (Sigma-Aidrich, United States) coupling reagent to identifying and characterizing the epitopes. d. In Dot-blot assay, 1 μg of each peptide or peptide–BSA was spotted onto nitrocellulose membranes (AE99, Schleicher & Schuell, Inc., Germany). SMCC-BSA was used as the negative control. After being incubated by 11H2 mAb or ASFV positive serum and washed by PBST, the membranes were incubated with horseradish peroxidase (HRP)conjugated goat anti-mouse or anti-pig IgG (H + L) and visualized. In peptide-based ELISA, 96-well plates were coated with 5 μg/ml peptide or peptide–BSA conjugates (100 μl/well) in 0.05 M CBS buffer. After being incubated by 11H2 mAb or ASFV positive serum and washed by PBST, the membranes were incubated with horseradish peroxidase (HRP)conjugated goat anti-mouse or anti-pig IgG (H + L) and visualized by TMB. The OD values of each well were measured at 450 nm using an ELISA microplate reader. The structure and epitopes of the CD2v extracellular domain were illustrated by PyMol software.

### Sequence datasets and conservation analysis

To assess the conservation of the identified epitopes, 17 CD2v protein sequences obtained from NCBI were compared with Cluaster, five of which are endemic Chinese strains. A phylogenetic tree for CD2v was constructed using MEAGA7.0 to determine the genetic relationship of 17 strains. To determine the conserved type of CD2v in different strains, 17 complete CD2v sequences were obtained from the PDB local comparison search tool (BLAST). SY-18 reference protein was used as the query template. Nonredundant protein sequence (NR) databases were selected for the search, including those from GenBank, PDB and SwissProt. The sequences were aligned by ClustalX. The results are displayed with Jalview.

## Results

### Purification and characterization of the recombinant CD2v protein

**Figure 3:**
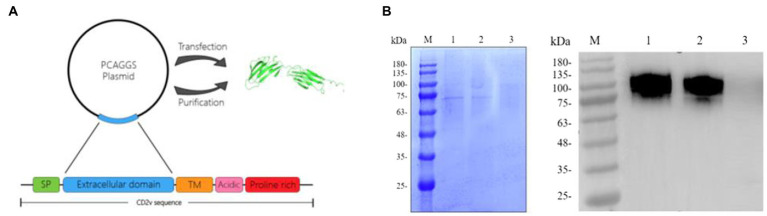
Preparation and purification of recombinant CD2v protein. **(A)** Preparation of the recombinant protein. The recombinant CD2v protein was sequenced to select the extracellular domain of the CD2v protein. The extracellular domain of the CD2v gene was ligated to the PCAGGS vector and transfected into 293F cells to express the recombinant protein. The protein structure diagram in the figure was predicted by AlphaFlod2 software. **(B)** Purified and unpurified CD2v recombinant proteins were analyzed by sodium dodecyl sulfate–polyacrylamide gel electrophoresis (SDS–PAGE) and western blot (WB) analysis. M is the protein marker, Lane 1 was loaded with purified CD2v recombinant protein, and Lane 2 was loaded with 293F cell culture medium before purification. Lane 3 was loaded with normal 293F cell culture medium (Control). The molecular weight of the recombinant protein is approximately 75–100 kD.

The CD2v extracellular region sequence was cloned into the pCAGGS vector, and the recombinant plasmid was transfected into 293F cells for expressing (Figure 3A). According to the SDS-PAGE results, the component of purified protein was relatively single compared to the cell culture medium, indicating a higher purity of the recombinant protein. Furthermore, the purified protein was verified by western blotting with ASFV-positive serum (Figure 3B). The result showed that the recombinant protein was well recognized by the positive serum, indicating that the protein had a high antigenicity (Figure 3B). The dispersion of the recombinant protein was probably due to the high-level glycosylation of the protein.

### Generation and characterization of anti-CD2v mAbs

**Figure 4:**
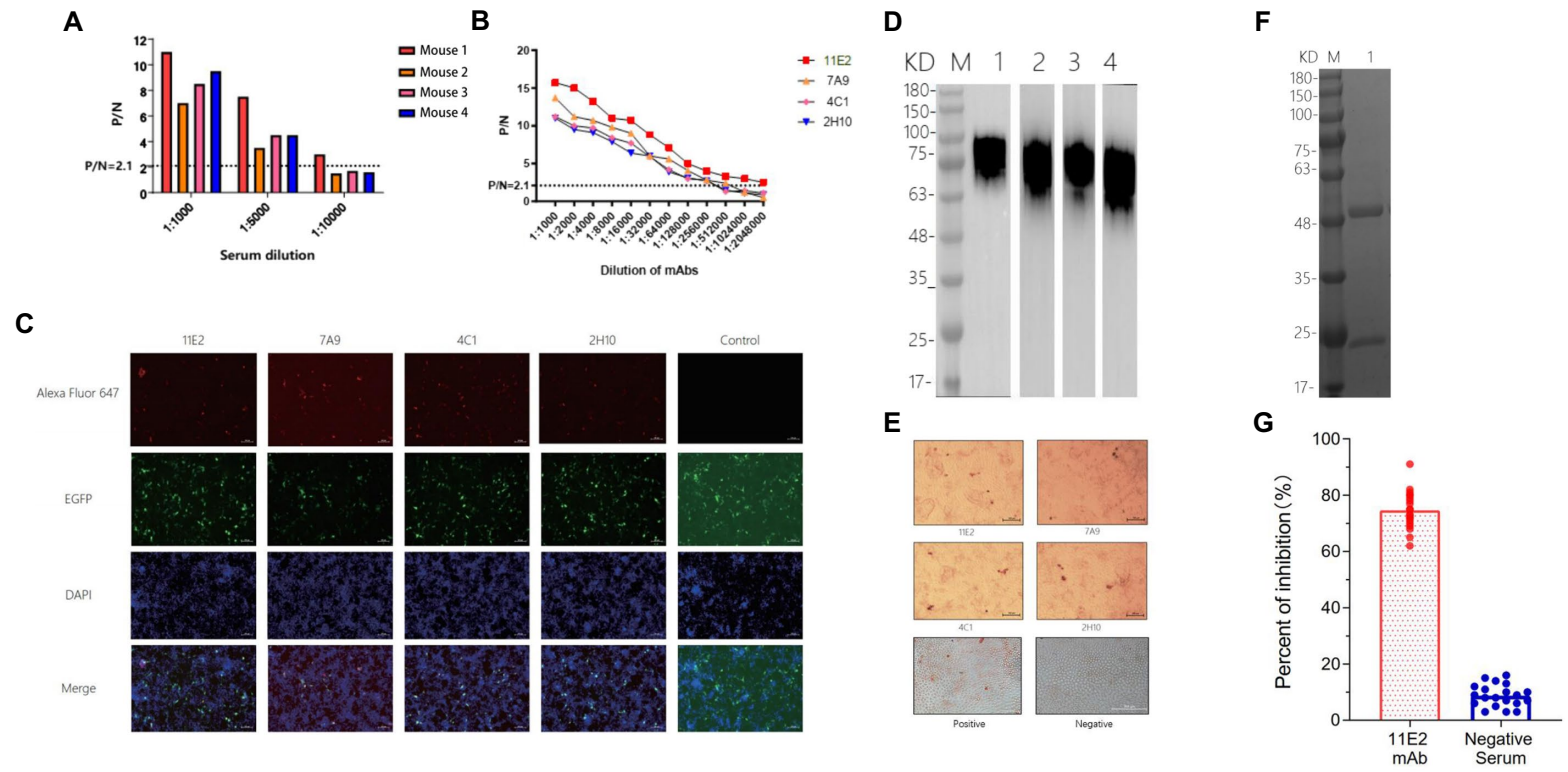
Characterization and identification of mAbs. **(A)** Determination of the serum titer of the mice before hyperimmunization by indirect ELISA. **(B)** Determination of the mAb titers. A P/G value greater than or equal to 2.1 is considered positive, and the corresponding maximum dilution is the titer of serum or mAbs. **(C)** Four mAbs were identified by IFA. The CD2v recombinant protein gene was linked to a vector containing the EGFP label and was transfected into 293 T cells. A monoclonal antibody was used as the primary antibody, and a goat anti-mouse Alexa Fluor 647 fluorescent antibody was used as the secondary antibody to display red fluorescence. DAPI was used as the nuclear staining fluid. The control group was used as a negative control. **(D)** Four mAbs were identified with CD2v recombinant protein by a western blot assay. M represents the protein marker, monoclonal antibody 11E2 was incubated in Lane 1, Lane 2 is 7A9, Lane 3 is 4C1 and Lane 4 is 2H10. **(E)** The reactivity of the mAbs with ASFV HLJ/18 strain. Scale bars, 200 μm. **(F)** SDS–PAGE analysis of purified monoclonal antibody 11E2. **(G)** CD2v mAbs evaluation by the blocking enzyme-linked immunosorbent assay. The African swine fever positive serum was serially diluted for 1:10 (*n* = 20).

Indirect ELISA was used to detect antibody levels in the mice serum before fusion. The results showed that the maximum serum titer of Mouse1 was over 1:10000 (Figure 4A). Four mAb hybridoma cell lines (11E2, 7A9, 4C1 and 2H10) were screened by examining the cell fusion between spleen cells from Mouse 1 and mouse myeloma (SP2/0) cells. The reactivity and affinity between the mAbs and protein were determined by ELISA. The results showed that the four mAbs had high titers, with titers of 1:2048,000, 1:512,000, 1:256,000, and 1:256,000, respectively, and 11E2 had the best affinity (Figure 4B). Transfection of CD2v plasmid into 293 T cells followed by identification of the reactivity of the monoclonal antibody with the natural CD2v protein by IFA. The results showed that all four mAbs could bind specifically to the protein and produce a red fluorescence signal (Figure 4C). To determine whether the four mAbs could react with the natural virus protein, an IPMA test was carried out, and the results showed that all mAbs had specific reactions with the virus (Figure 4E). To determine the epitopes that were recognized by the four mAbs, a western blot test with CD2v protein was carried out (Figure 4D). The results showed that all four mAbs could bind to the CD2v protein, indicating that all four mAbs recognized linear epitopes. Four strains of mAbs were IgG2a with κlight chain (Table 1). We selected the mAb 11E2 with the highest titer for purification for further validation (Figure 4F). Meanwhile, the inhibition rate of 11E2 monoclonal antibody reached about 77% in the blocking experiment with ASFV-positive pig serum. This result indicated that the antibody was the competitive antibody in the positive serum, and the bound epitope and target might be the dominant epitope (Figure 4G).

**Table 1:** Identification of the subclasses of CD2v monoclonal antibodies.

	Monoclonal antibodies			
	11E2	7A9	4C1	2H10
Ig subclass	IgG2a	IgG2a	IgG2a	IgG2a
Light chain type	κ	κ	κ	κ

### Epitope mapping of anti-CD2v mAbs

To determine the binding epitopes of the four mAbs, we transferred plasmid containing truncated gene into 293 T cells for identification by IFA. The success of transfection was reflected by using the EGFP-C vector, which contains the EGFP label. Green fluorescence was observed for the EGFP labeling after successful transfection. A reaction that showed red fluorescence was positive (Figure 2A). The results indicated that all mAbs reacted with plasmids C1, C2, C2-1, C2-2, C2-3, C2-3-1, and C2-3-2 (Figure 2A). The amino acid sequences of the epitopes are shown in Table S1. The results indicated that C2, C2-3, and C2-3 could react well with the four mAbs, but the other groups did not react. In the C2-3 group, a reaction with C2-3-2 was observed, but not with C2-3-1 (Figures 2B–E). C2-3-1 and C2-3-2 overlapped some amino acids (Figure 2A), thus the epitope recognized by the 4 mAbs was finally determined to be ^154^SILEYNW^160^ (Figures 2A–E). In order to confirm the key amino acids at the binding site of monoclonal antibody, in this experiment, the peptides synthesis and alanine scanning were the further confirmed method. The verification method was peptide-based Elisa and Dot-blot (Figures 2F,G). According to the positive and negative control, the result of reactivity between peptides and mAb (or positive serum) is reliable. These results indicated that 154SILE157 was the smallest amino acid sequence recognized by the monoclonal antibody, and it had good reactivity with ASFV positive sera, which maintained the antigenicity of the epitope (Figures 2F,G).

To determine the position of the epitope in the spatial structure of the protein, we used software to show the spatial structure of the protein and mark the position of the epitope (Figure 2H).

### Conserved analysis of epitopes in different viral strains

**Figure 5:**
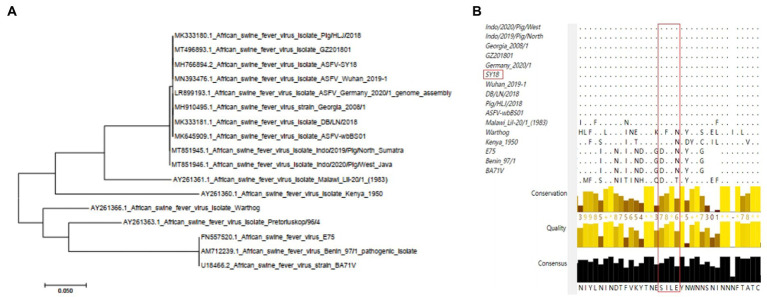
Conservation analysis of CD2v extracellular domains and epitopes in different representative ASFV strains. **(A)** Phylogenetic trees were established for the phylogenetic analysis of 17 representative ASFV strains. The NJ tree was established by MEGA7.0. **(B)** Analysis of the conservation of epitopes in 17 representative strains by ClustalX.

To evaluate the conserved nature of epitope sequences in different strains, 17 epidemic strains were selected for sequence conservation analysis and phylogenetic tree construction. The phylogenetic tree results showed that the CD2v sequences of the five strains prevalent in China were highly consistent with those of Georgia 2008/1 and several Chinese epidemic strains, such as Wuhan 2019, HLJ 2018 and others (Figure 5A). Multiple alignment of amino acid sequences showed that the same ^154^SILE^157^ epitope was conserved in strain Georgia 2008/1 and other Chinese epidemic strains but was basically different in strains Kennya1950, E75, BA71 V, Benin97/1 and Warthog (Figure 5B). The results showed that the ^154^SILE^157^ epitope is highly conserved in the domestic epidemic strains and Georgia/2008 strains, but there was little overlap with other strains.

## Discussion

ASFV is a virus, which has the large genome and numerous structural proteins. Finding the proper antigenic target proteins is the key to minimize the risk of ASFV (Gaudreault et al., 2020). Numerous studies have demonstrated that CD2v is a crucial antigen in the ASFV-induced protective immune response (Pérez-Núñez et al., 2015). Meanwhile, some researchs has revealed that CD2v could induce the immune response in pigs that were immunized with exogenously-expressed CD2v protein (Kay-Jackson et al., 2004). In the side of the protein construction, the extracellular domain of CD2v contains two immunoglobulin-like domains, which are remarkably similar to the extracellular domain of the CD2 protein (Recny et al., 1990). This also suggests the possibility of immune function of CD2v. More constructively, some studies have shown that T-cell epitopes are screened in the extracellular domain of CD2v (Burmakina et al., 2019; Mima et al., 2020). However, the CD2v protein, as the crucial functional antigen, there are few studies on B-cell epitopes. Only several B-cell epitopes have been reported recently (Jia et al., 2022; Ren et al., 2022). Therefore, the extracellular domain of CD2v was selected as the immunogen of immunized animals for the expression and purification procedures in this study. MAbs were screened by hybridoma cell technology to identify B-cell epitopes (Figure 1).

CD2v protein contains a large number of glycosylation sites, complete glycosylation modification cannot be achieved by prokaryotic expression system (Portolano et al., 2014). Therefore, the 293F mammalian eukaryotic expression system was selected for the expression of recombinant CD2v protein in this study (Figure 3A). To avoid redundant purification tag sequences affecting the immunogenicity of CD2v, we purified the protein by ion exchange chromatography (Figure 3B). The SDS-PAGE results showed that the recombinant protein was approximately 75–100 KD, much larger than the predicted molecular weight of approximately 45 KD, indicating that the recombinant CD2v protein was highly glycosylated. The purified CD2v protein bands were relatively homogeneous and responded well to swine positive sera (Figures 1A, 3B).

Additionally, we were able to successfully produce four monoclonal antibodies with high titer by the CD2v recombinant protein expressed in the 293F eukaryotic system, which had excellent immunogenicity (Figure 4B). Results from western blotting showed that all 4 mAbs reacted specifically with the denatured CD2v protein, indicating that the mAbs recognized linear B cell epitopes (Figure 4D). Meanwhile, IFA and IPMA showed that all 4 mAbs have effective reactivity with natural proteins and viruses (Figures 4C,F), implying that these mAbs could be used in the diagnosis of ASFV.

Epitopes are key elements in determining the antigenicity of viral structural proteins and inducing the humoral immune response (Gottlieb and Ben-Yedidia, 2014; Grifoni et al., 2021).Particularly, the protective antigen of ASFV is still unknown. Screening of B-cell epitopes is a critical step in the development of vaccines (Ge et al., 2018). In previous studies, CD2v protein epitopes were predicted using bioinformatics techniques and four T cell epitopes were identified (Burmakina et al., 2019). And in another study, we also have identified several epitopes (Ren et al., 2022). But in these epitopes, the crucial aminos are not clear and the conservation of the sequences has not been identified. And in this study, the specific shortest amino acid sequence of this epitope has been accurately identified for the first time. Compared with previous studies, our study characterized the key amino acids of the epitopes recognized by mAbs, and carried out sequence comparison and phylogenetic tree analysis between classical and endemic strains of ASFV, and reached a conclusion that the epitope is relatively conserved among endemic strains in China. These were not revealed in previous studies. Meanwhile, due to the abundant glycosylation modifications of CD2v, traditional identification of overlapping peptides is not applicable in this study as peptides expressed by E. coli or directly synthesised do not have glycosylation modifications (Rodríguez et al., 1993; Malogolovkin and Kolbasov, 2019). In this study, a series of recombinant plasmids were constructed and transfected with human embryonic kidney 293 T cells. 293 T cells are a eukaryotic expression system and the constructed plasmids can be glycosylation modified after transfection to avoid the recognition barrier of unglycosylated epitopes (Portolano et al., 2014). Ultimately a novel B-cell epitope ^154^SILEYNW^160^ was identified (Figures 2A–E). Furthermore, we identified several crucial amino acid residues, 154SILE157, by peptide synthesis and alanine scanning (Figures 2F,G). We predicted the protein structure of the CD2v protein and information on the spatial location of the identified epitopes by PyMol. Sequence alignment showed that the ^154^SILE^160^ epitope was only conserved in the Chinese endemic strains and gerogia 2008/1, suggesting that this epitope is an important feature of this branch of ASFV and can be used for differential diagnosis of different strains. Considering that there is no cross-protection between different strains of ASFV, this epitope may be one of the candidate vaccine antigens for the strains prevalent in China. Meanwhile, in terms of the protection of the epitope, the inhibition rate of 11E2 monoclonal antibody recognizing this epitope against ASFV-positive pig sera reached 77%, which illustrates that the antibodies induced by this epitope occupy a dominant position in the serum of ASFV positive pigs (Figure 4G). The good response of 11E2 mAbs recognizing this epitope to the virus also illustrates the preponderant exposure state of this epitope on the native virus surface in the IPMA experiments, which means that this epitope may have a good positional advantage for binding and recognition (Figure 4E). What is more convincing is that the Dot-Blot and Elisa experiments of the epitope showed a good reaction effect with the positive serum, which directly demonstrated the good binding and reactivity of this epitope with ASFV pig positive serum (Figures 2F,G). These results show that epitope also have a fair effect on protection. In summary, we report four monoclonal antibodies targeting the extracellular region of the ASFV CD2v protein and a linear B-cell epitope (154SILE157). All four mAbs with the same epitope react with ASFV and contribute to further studies of ASFV structure and function. The epitopes specific to the prevalent Chinese strains will be useful for vaccine development and differential diagnosis of different strains. In recent studies, the CD2v deletions strains were isolated. Therefore, the diagnostic application of this epitope may also have important significance and potential in distinguishing CD2v deletions from other strains (Sun et al., 2021).

## Data availability statement

The datasets presented in this study can be found in online repositories. The names of the repository/repositories and accession number(s) can be found in the article/Supplementary material.

## Ethics statement

The animal study was reviewed and approved by the Key Laboratory of Animal Immunology, Henan Academy of Agricultural Sciences.

## Author contributions

PD, SL, and GZ conceived and designed the research. SL, DR, YD, ML, YC, SW, XS, ZC, and RL performed the experiments. SL performed data analysis. PD and SL drafted the manuscript. All authors contributed to the article and approved the submitted version.

## Funding

This work was funded by the National Natural Science Foundation of China (31941001).

## Conflict of interest

The authors declare that the research was conducted in the absence of any commercial or financial relationships that could be construed as a potential conflict of interest.

## Publisher’s note

All claims expressed in this article are solely those of the authors and do not necessarily represent those of their affiliated organizations, or those of the publisher, the editors and the reviewers. Any product that may be evaluated in this article, or claim that may be made by its manufacturer, is not guaranteed or endorsed by the publisher.

## Footnotes

^1^ https://www.oie.int/en/animal-health-in-the-world/animal-diseases/african-swine-fever
