# Supplementary material for: Development and characterization of monoclonal antibodies against the extracellular domain of African swine fever virus structural protein, CD2v
Source: Front Microbiol. 2022 Nov 18;13:1056117. doi: 10.3389/fmicb.2022.1056117 (PMC9716131; doi:10.3389/fmicb.2022.1056117)
Supplement: Supplementary file 1 [file Table_1.docx]

| Table 1 Amino acid sequence of truncated fragments | |
| --- | --- |
| Designation | Designation |
| C1 | IDYWVSFNKTIILDSNITNDNNDINGVSWNFFNNSF |
| C2 | NTLATCGKAGNFCECSNYSTSIYNITNNCSLTIFPHNDV  FDTTYQVVWNQIINYTIKLLTPATPPNITYNCTNFLIT  CKKNNGTNTNIYLNINDTFVKYTNESILEYNWNNSNIN  NFTATCIINNTISTSNETTLINCTYLTLSSNYFYTFFKL Y |
| C2-1 | NTLATCGKAGNFCECSNYSTSIYNITNNCSLTIFPHN  DVFDTTYQVVWNQIINYTIKLLTPA TPPNITYNCTN  FLITCKK |
| C2-2 | ITNNCSLTIFPHNDVFDTTYQVVWNQIINYTIKLLTPATP |
| C2-3 | TNIYLNINDTFVKYTNESILEYNW |
| C2-3-1 | TNIYLNINDTFVKYTNE |
| C2-3-2 | FVKYTNESILEYNW |
